# Carbon monoxide poisoning following a ban on household use of raw coal, Mongolia

**DOI:** 10.2471/BLT.22.289232

**Published:** 2023-05-18

**Authors:** Tsetsegee Sambuu, Gerelmaa Gunsmaa, Tumen Ulzii Badarch, Yerkyebulan Mukhtar, Masao Ichikawa

**Affiliations:** a Graduate School of Comprehensive Human Sciences, University of Tsukuba, 1-1-1 Tennodai, Tsukuba, Ibaraki 305-8577, Japan.; b Department of Statistics and Surveillance, National Trauma and Orthopaedic Research Centre, Ulaanbaatar, Mongolia.; c Department of Epidemiology and Biostatistics, Mongolian National University of Medical Sciences, Ulaanbaatar, Mongolia.; d Department of Global Public Health, Faculty of Medicine, University of Tsukuba, Ibaraki, Japan.

## Abstract

**Objective:**

To examine trends in the incidence of carbon monoxide poisoning before and after a ban on domestic use of raw coal in Ulaanbaatar, Mongolia.

**Methods:**

Using injury surveillance data and population estimates, we calculated the incidence per 100 000 person-years of fatal and non-fatal domestic carbon monoxide poisoning before (May 2017 to April 2019) and after (May 2019 to April 2022) the ban in May 2019. We analysed data by age and sex, and compared areas not subjected to the ban with districts where domestic use of raw coal was banned and replaced with refined coal briquettes.

**Findings:**

We obtained complete data on 2247 people with carbon monoxide poisoning during the study period in a population of around 3 million people. In districts with the ban, there were 33 fatal and 151 non-fatal carbon monoxide poisonings before the ban, and 91 fatal and 1633 non-fatal carbon monoxide poisonings after the ban. The annual incidence of poisoning increased in districts with the ban, from 7.2 and 6.4 per 100 000 person-years in the two 12-month periods before the ban to 38.9, 42.0 and 40.1 per 100 000 in the three 12-month periods after the ban. The incidence of poisoning remained high after the ban, despite efforts to educate the public about the correct use of briquettes and the importance of ventilation. The incidence of carbon monoxide poisoning also increased slightly in areas without the ban.

**Conclusion:**

Efforts are needed to investigate heating practices among households using briquettes, and to determine factors causing high carbon monoxide concentrations at home.

## Résumé

**Objectif:**

Examiner les tendances en ce qui concerne la fréquence des intoxications au monoxyde de carbone avant et après une interdiction de l'usage domestique du charbon brut à Oulan-Bator, en Mongolie.

**Méthodes:**

En nous fondant sur les données de surveillance des traumatismes et les estimations de population, nous avons calculé la fréquence, pour 100 000 années-personnes, des intoxications domestiques mortelles et non mortelles au monoxyde de carbone avant (de mai 2017 à avril 2019) et après (de mai 2019 à avril 2022) l'interdiction survenue en mai 2019. Nous avons analysé les données par âge et sexe, et nous avons comparé les régions non concernées par cette interdiction avec les districts où l'usage domestique du charbon brut a été proscrit et remplacé par des briquettes de charbon raffiné.

**Résultats:**

Nous avons obtenu des informations complètes sur 2247 individus ayant subi une intoxication au monoxyde de carbone durant la période couverte par l'étude au sein d'une population d'environ 3 millions de personnes. Dans les districts concernés par l'interdiction, 33 intoxications mortelles et 151 intoxications non mortelles ont eu lieu avant que le charbon brut ne soit prohibé, contre 91 intoxications mortelles et 1633 intoxications non mortelles après. La fréquence annuelle des intoxications a augmenté dans les districts concernés par l'interdiction, passant de 7,2 et 6,4 pour 100 000 années-personnes au cours des deux périodes de 12 mois précédant l'interdiction à 38,9, 42,0 et 40,1 pour 100 000 années-personnes au cours des trois périodes de 12 mois suivant l'interdiction. Cette fréquence est restée élevée après l'interdiction, en dépit des efforts menés pour sensibiliser le public à l'utilisation appropriée des briquettes et à l'importance de l'aération. La fréquence des intoxications au monoxyde de carbone s'est en outre légèrement accentuée dans les régions non concernées par l'interdiction.

**Conclusion:**

Des efforts sont nécessaires afin d'enquêter sur les pratiques des ménages en matière d'utilisation des briquettes et de déterminer les facteurs à l'origine des fortes concentrations de monoxyde de carbone dans les habitations.

## Resumen

**Objetivo:**

Analizar las tendencias en la incidencia de intoxicación por monóxido de carbono antes y después de una prohibición de uso doméstico de carbón sin procesar en Ulaanbaatar, Mongolia.

**Métodos:**

Utilizando datos sobre vigilancia de lesiones y estimaciones de población, calculamos la incidencia por 100 000 personas-años de la intoxicación mortal y no mortal por monóxido de carbono doméstico antes (de mayo de 2017 a abril de 2019) y después (de mayo de 2019 a abril de 2022) de la prohibición realizada en mayo de 2019. Analizamos los datos por edad y sexo, y comparamos las zonas en las que no existía prohibición con aquellos distritos en los que se había prohibido el uso doméstico de carbón sin procesar y se había reemplazado por briquetas de carbón refinado.

**Resultados:**

Obtuvimos datos detallados sobre 2247 personas con intoxicación por monóxido de carbono durante el periodo de estudio en una población de 3 millones de personas aproximadamente. En determinados distritos y antes de la prohibición, hubo 33 intoxicaciones mortales por monóxido de carbono y 151 intoxicaciones no mortales. Después de la prohibición, hubo 91 intoxicaciones mortales por monóxido de carbono y 1633 intoxicaciones no mortales. En distritos en los que existía prohibición, la incidencia anual de intoxicación aumentó de 7,2 y 6,4 por 100 000 personas-años en los dos periodos de 12 meses anteriores a la prohibición, a 38,9, 42,0 y 40,1 por 100 000 en los tres periodos de 12 meses posteriores a la prohibición. La incidencia de intoxicación continúa siendo elevada tras la prohibición, a pesar de los esfuerzos por formar a los ciudadanos acerca del uso correcto de las briquetas y de la importancia de la ventilación. La incidencia de intoxicación por monóxido de carbono también aumentó ligeramente en zonas en las que no existía prohibición.

**Conclusión:**

Hay que aunar esfuerzos con el fin de investigar las prácticas de calefacción en aquellos hogares que utilizan briquetas, y de determinar los factores que causan las elevadas concentraciones de monóxido de carbono en el hogar.

## ملخص

**الغرض:**

بحث اتجاهات حدوث التسمم بأول أكسيد الكربون قبل حظر الاستخدام المحلي للفحم الخام وبعده، في أولانباتور بمنغوليا.

**الطريقة:**

باستخدام بيانات مراقبة الإصابات وتقديرات السكان، قمنا بحساب معدل حدوث التسمم المحلي بأول أكسيد الكربون المميت، وغير المميت، لكل 100000 شخص في العام، قبل (مايو/أيار 2017 إلى أبريل/نيسان 2019)، وبعد (مايو/أيار 2019 إلى أبريل/نيسان 2022) الحظر في مايو/أيار 2019. قمنا بتحليل البيانات حسب العمر والجنس، ومقارنة المناطق غير الخاضعة للحظر مع المناطق التي تم فيها حظر الاستخدام المحلي للفحم الخام، واستبداله بقوالب الفحم المكرر.

**النتائج:**

حصلنا على بيانات كاملة عن 2247 شخصًا أصيبوا بتسمم بأول أكسيد الكربون خلال فترة الدراسة في عدد سكان يبلغ حوالي 3 ملايين شخص. في المناطق التي الحظر فيها كان هناك 33 حالة تسمم مميت، و151 حالة تسمم غير مميت بأول أكسيد الكربون قبل الحظر، و91 حالة تسمم مميت، و1633 حالة تسمم غير مميت بأول أكسيد الكربون بعد الحظر. زاد معدل حدوث التسمم السنوي في المقاطعات الخاضعة للحظر من 7.2 و6.4 لكل 100000 شخص في العام، في فترتي الاثني عشر شهرًا قبل الحظر إلى 38.9 و42.0 و40.1 لكل 100000 في فترات الاثني عشر شهرًا الثلاث بعد الحظر. ظلت معدل حدوث التسمم مرتفعًا بعد الحظر، بالرغم من الجهود المبذولة لتوعية الجمهور حول الاستخدام الصحيح للقوالب وأهمية التهوية. كما زاد معدل حدوث التسمم بأول أكسيد الكربون بشكل طفيف في المناطق التي لا تخضع للحظر.

**الاستنتاج:**

هناك حاجة إلى بذل جهود للتحقيق في ممارسات التدفئة بين الأسر التي تستخدم قوالب الفحم الحجري، ولتحديد العوامل التي تسبب تركيزات عالية من أول أكسيد الكربون في المنزل.

## 摘要

**目的:**

旨在研究蒙古乌兰巴托禁止家庭使用原煤前后一氧化碳中毒发生率的趋势。

**方法:**

使用伤害监测数据和人口估计,我们计算了 2019 年 5 月禁令实施之前(2017 年 5 月至 2019 年 4 月)和之后(2019 年 5 月至 2022 年 4 月)每 10 万人每年致命和非致命家庭一氧化碳中毒的发生率。我们按年龄和性别对数据进行了分析,并将未受禁令约束的地区与禁止家庭使用原煤并代之以精煤块的地区进行了比较。

**结果:**

在研究期间,在约 300 万人中,我们获得了 2,247 名一氧化碳中毒患者的完整数据。在受禁令约束的地区,在禁令实施之前发生了 33 起致命和 151 起非致命一氧化碳中毒事件,禁令实施之后发生了 91 起致命和 1,633 起非致命一氧化碳中毒事件。受禁令约束的地区的年中毒发生率从禁令实施前两个 12 个月期间的每 10 万人每年 7.2 起和 6.4 起增加到禁令实施后三个 12 个月期间的每 10 万人每年 38.9 起、42.0 起和 40.1 起。尽管公众已被普及如何正确使用煤块以及确保通风的重要性,但禁令实施后,中毒的发生率仍然很高。在未受禁令约束的地区,一氧化碳中毒的发生率也略有上升。

**结论:**

需要调查使用煤块的家庭的取暖方式,并确定造成家庭中一氧化碳浓度较高的因素。

## Резюме

**Цель:**

Изучить тенденции в частоте случаев отравления угарным газом до и после запрета на бытовое использование сырого угля в г. Улан-Батор, Монголия.

**Методы:**

На основании данных системы эпидемиологического мониторинга травматизма и демографических оценок было рассчитано количество случаев бытового отравления угарным газом со смертельным и несмертельным исходом на 100 000 человеко-лет до (с мая 2017 года по апрель 2019 года) и после (с мая 2019 года по апрель 2022 года) введения запрета в мае 2019 года. Данные анализировали по возрасту и полу и сравнивали районы, на которые запрет не распространялся, с районами, где бытовое использование сырого угля было запрещено и заменено угольными брикетами.

**Результаты:**

За исследуемый период были получены полные данные о 2247 людях с отравлением угарным газом в популяции численностью около 3 миллионов человек. В районах с введенным запретом было зарегистрировано 33 случая отравления угарным газом со смертельным исходом и 151 случай отравления угарным газом без смертельного исхода до введения запрета, а также 91 случай отравления угарным газом со смертельным исходом и 1633 случая отравления угарным газом без смертельного исхода после введения запрета. Ежегодные показатели частоты отравлений увеличились в районах с введенным запретом с 7,2 и 6,4 на 100 000 человеко-лет в течение двух 12-месячных периодов до введения запрета до 38,9; 42,0 и 40,1 на 100 000 в течение трех 12-месячных периодов после введения запрета. Несмотря на мероприятия по информированию населения о правильном использовании брикетов и важности вентиляции, показатели частоты отравлений оставались высокими и после введения запрета. Частота случаев отравления угарным газом также несколько возросла в районах, где запрет не действует.

**Вывод:**

Необходимо предпринять усилия для изучения способов отопления домохозяйств с использованием брикетов и определить факторы, обуславливающие высокую концентрацию угарного газа в домах.

## Introduction

Air pollution is a long-lasting public health issue that causes premature death globally. An annual estimated number of deaths associated with ambient and household air pollution is 4.2 million and 2.5 million, respectively.^1^ Most deaths occur in low- and middle-income countries, mainly in South-East Asia and Western Pacific Regions.^1^ Mongolia is included in Western Pacific Region, and its capital, Ulaanbaatar, is one of the world’s most polluted cities.^2^

In Ulaanbaatar, raw coal is commonly used for domestic heating and cooking.^3^ Because the average temperature is below or close to zero degrees Celsius for 8 months of the year (from mid-September to mid-May), heating requires large amounts of raw coal, especially in Ulaanbaatar where approximately half the households (208 049 of 414 292 houses) are traditional tent-like dwellings (called *ger*) or simple detached houses without central heating.^4^ The domestic use of raw coal accounts for about 10% of the 11 million tons of raw coal used in Ulaanbaatar,^5^ but it causes an estimated 80% of ambient concentrations of fine particulate matter PM2.5 in the air in Ulaanbaatar.^6^^,7^

To take action on air pollution, the Mongolian government initiated the National Programme for Reducing Air and Environmental Pollution in 2017, where the introduction of refined coal briquettes was planned for the first time.^8^ In February 2018, the government issued a resolution to ban the domestic use of raw coal,^9^ and started publicity campaigns to inform the public that raw coal use would be banned at home, and briquettes would be supplied as alternative heating energy.^10^^,^^11^ In May 2019, the ban became effective in six of the nine districts in Ulaanbaatar.^12^ In October 2019, when the cold season started and heating was required, the incidence of carbon monoxide poisoning at home suddenly increased.^13^ Authorities suspected that the incomplete combustion of briquettes was causing a toxic level of carbon monoxide inside the houses.

In response to the sudden increase in domestic carbon monoxide poisoning, the government began a programme of awareness campaigns and home visits for inspections, which are still ongoing.^14^ An awareness campaign was conducted through radio, television and social networking sites, informing the public of the danger of carbon monoxide poisoning, the proper use of briquettes and furnaces, and the importance of ventilation to prevent carbon monoxide poisoning.^15^^–^^18^ Home visits aimed to check the conditions of furnaces and chimneys, cleaning and repairing them if necessary, at no cost for needy families, while carbon monoxide sensors were provided free of charge to all households in Ulaanbaatar.^14^^,^^19^ To our knowledge, there is scant evidence on the extent to which domestic carbon monoxide poisoning increased after the introduction of the raw coal ban, and whether poisonings decreased after the provision of countermeasures against carbon monoxide poisoning. We therefore aimed to examine the trend in the incidence of domestic carbon monoxide poisoning in Ulaanbaatar before and after the ban.

## Methods

### Study setting

Ulaanbaatar, the capital city of Mongolia, consists of nine districts with nearly half of the country’s total population of 3.4 million people. In May 2019, the government banned domestic raw coal use in six districts of Ulaanbaatar, namely Bayangol, Bayanzurkh, Chingeltei, Khan-Uul, Songinokhairkhan and Sukhbaatar. These districts are adjacent to each other in the city centre. Of the remaining districts, Nalaikh is situated on the outskirts of the city, while Bagakhangai and Baganuur, although officially part of the city, are situated roughly 100 km away from the city centre. The raw coal ban was extended to Nalaikh in February 2022. This study was approved by the research ethics committee of the Faculty of Medicine at the University of Tsukuba, and the National Trauma and Orthopaedic Research Centre.

### Data

We obtained data on carbon monoxide poisoning from the National Trauma and Orthopaedic Research Centre, which manages injury mortality and morbidity data from the National Institute of Forensic Science and all health facilities across the country in the national surveillance system (including national, provincial and district hospitals; family health centres; and private hospitals). Injury data are recorded with International Statistical Classification of Diseases and Related Health Problems (ICD) 10th Revision codes, using code T58 for the toxic effect of carbon monoxide. Carbon monoxide poisoning is typically diagnosed based on symptoms, anamnesis, physical examinations and blood tests.^20^ Patients with a carboxyhaemoglobin level of 20% or above in their blood (and pregnant women, children and older patients with a level of 10% or above) are hospitalized or transferred to the national or provincial hospitals. Patients with a carboxyhaemoglobin level below 20% are monitored at district hospitals or family health centres. A patient’s sex is usually determined through an external examination of their body characteristics during treatment.

We obtained data on patients with fatal and non-fatal carbon monoxide poisoning occurring between May 2017 and April 2022. Data included patient’s age, sex, place of residence, date and place of carbon monoxide poisoning, type of health facility attended and whether carbon monoxide poisoning was fatal. We obtained population data for each district of Ulaanbaatar and each province between 2017 and 2022 from the national statistical office. Since monthly population estimates were unavailable, we obtained mid-year population estimates by sex in each district and each province. We then interpolated monthly population estimates, using the tempdisagg package of R version 3.6.2 software (R Foundation, Vienna, Austria).

### Analysis

We analysed data on carbon monoxide poisoning that happened at home. First, we described the sex and age distribution of the patients with fatal and non-fatal poisonings before the ban from May 2017 to April 2019, and after the ban from May 2019 to April 2022. We examined changes in the patients’ profiles in three settings: (i) the six districts of Ulaanbaatar subjected to the raw coal ban; (ii) the three districts of Ulaanbaatar not subjected to the ban; and (iii) the rest of the country outside Ulaanbaatar. We then graphically inspected the trend in the monthly number of carbon monoxide poisonings per 100 000 person-years (monthly incidence rate) from May 2017 to April 2022 in the three settings. The numerator was the sum of the number of fatal and non-fatal carbon monoxide poisonings that occurred each month. We summed the number of fatal and non-fatal carbon monoxide poisonings because the monthly number of fatal poisonings was small. The denominator was the monthly population estimates divided by 12, multiplied by 100 000 to generate the rate per 100 000 person-years. 

Next, we calculated the annual incidence of fatal and non-fatal carbon monoxide poisoning in the three settings in 12-month periods before the ban (May 2017 to April 2018; May 2018 to April 2019) and after the ban (May 2019 to April 2020; May 2020 to April 2021; May 2021 to April 2022). We aimed to examine the extent to which the incidence of carbon monoxide poisoning increased after the ban, and whether the incidence decreased due to awareness-raising measures taken to prevent carbon monoxide poisoning. The numerator of the rate was the sum of the number of fatal or non-fatal carbon monoxide poisonings that occurred in the respective period. The denominator was the sum of monthly population estimates divided by 12 in the respective period, multiplied by 100 000 to generate the rate per 100 000 person-years.

The incidence rates calculated may be considered as incidence proportions (or cumulative incidence) because accurate person-time information was not available to calculate the incidence rates. However, the numerator (patients with carbon monoxide poisoning) was very small relative to the denominator (population at risk of carbon monoxide poisoning), and most of the population was not affected by carbon monoxide poisoning. Therefore, the incidence rates in this study were comparable to person-time rates.

Finally, we examined annual trends in the incidence of poisoning after the ban using incidence rate ratios and their 95% confidence interval (CI). We calculated the ratio of incidence of fatal or non-fatal carbon monoxide poisoning in the three 12-month periods after the raw coal ban (May 2019 to April 2020; May 2020 to April 2021; May 2021 to April 2022) relative to the 24-month period before the ban (May 2017 to April 2019). We used Stata (17.0, StataCorp LLC, College Station, United States of America) for this calculation.

## Results

During the study period, there were 2304 carbon monoxide poisonings. Among them, 17 patients lived outside Ulaanbaatar but were hospitalized in Ulaanbaatar. Since we were unsure whether these carbon monoxide poisonings occurred in Ulaanbaatar or outside, we excluded these patients from the analysis. Moreover, the district of residence was not recorded for 40 patients who lived in Ulaanbaatar. We assumed that most of these patients came from the districts with the ban, because around 95% (1 466 431 people) of the 1 539 252 residents of Ulaanbaatar lived in the districts with the ban. Since the analyses including and excluding these patients provided similar results, we report the results from the 2247 people with complete data (1321 female and 926 male). 

Across all areas, 224 people were diagnosed with carbon monoxide poisoning in the period before the ban (May 2017 to April 2019) and 2023 people in the period after the ban (May 2019 to April 2022). Table 1 shows the sex and age distribution of patients with fatal and non-fatal carbon monoxide poisoning before and after the ban. In Ulaanbaatar’s districts with the ban, there were 33 fatal and 151 non-fatal poisonings before the ban, and 91 fatal and 1633 non-fatal poisonings after the ban. The proportion of males and females with poisoning was similar before and after the ban for both fatal and non-fatal poisonings. Males accounted for a higher proportion of the fatal poisonings before (25 deaths, 75.8%) and after the ban (64 deaths, 70.3%), while females accounted for a higher proportion of non-fatal poisonings before (89 cases, 58.9%) and after the ban (1016 cases, 62.2%). Their age distribution appeared to be similar before and after the ban when fatal and non-fatal poisonings were combined. However, the proportion of adults (aged 20 years or older) increased disproportionately among fatal poisonings from 69.7% (23 adults) before the ban to 85.7% (78 adults) after the ban. 

**Table 1:** Proportion of fatal and non-fatal carbon monoxide poisoning by sex and age group before (May 2017 to April 2019) and after (May 2019 to April 2022) the raw coal ban in Ulaanbaatar, Mongolia

Variable	Ulaanbaatar districts with the ban						Ulaanbaatar districts without the ban						Areas outside Ulaanbaatar				
	No. (%) of fatal poisonings			No. (%) of non-fatal poisonings			No. (%) of fatal poisonings			No. (%) of non-fatal poisonings			No. (%) of fatal poisonings			No. (%) of non-fatal poisonings	
	Before	After		Before	After		Before	After		Before	After		Before	After		Before	After
**Sex**																	
Male	25 (75.8)	64 (70.3)		62 (41.1)	617 (37.8)		0 (0.0)	1 (50.0)		1 (100.0)	14 (45.2)		2 (100.0)	17 (70.8)		20 (54.1)	103 (42.6)
Female	8 (24.2)	27 (29.7)		89 (58.9)	1016 (62.2)		0 (0.0)	1 (50.0)		0 (0.0)	17 (54.8)		0 (0.0)	7 (29.2)		17 (45.9)	139 (57.4)
**Age group, years**																	
0–4	3 (9.1)	4 (4.4)		0 (0.0)	15 (0.9)		0 (0.0)	0 (0.0)		0 (0.0)	0 (0.0)		0 (0.0)	0 (0.0)		2 (5.4)	20 (8.3)
5–9	3 (9.1)	3 (3.3)		0 (0.0)	6 (0.4)		0 (0.0)	0 (0.0)		0 (0.0)	0 (0.0)		0 (0.0)	0 (0.0)		6 (16.2)	17 (7.0)
10–14	3 (9.1)	4 (4.4)		0 (0.0)	19 (1.2)		0 (0.0)	0 (0.0)		0 (0.0)	0 (0.0)		0 (0.0)	3 (12.5)		2 (5.4)	14 (5.8)
15–19	1 (3.0)	2 (2.2)		8 (5.3)	109 (6.7)		0 (0.0)	0 (0.0)		0 (0.0)	4 (12.9)		0 (0.0)	0 (0.0)		3 (8.1)	26 (10.7)
20–29	5 (15.2)	7 (7.7)		46 (30.5)	419 (25.7)		0 (0.0)	0 (0.0)		0 (0.0)	13 (41.9)		0 (0.0)	4 (16.7)		5 (13.5)	56 (23.1)
30–39	7 (21.2)	13 (14.3)		36 (23.8)	393 (24.1)		0 (0.0)	0 (0.0)		0 (0.0)	5 (16.1)		0 (0.0)	4 (16.7)		5 (13.5)	42 (17.4)
40–49	3 (9.1)	16 (17.6)		27 (17.9)	325 (19.9)		0 (0.0)	2 (100.0)		1 (100.0)	8 (25.8)		0 (0.0)	8 (33.3)		2 (5.4)	25 (10.3)
50–59	5 (15.2)	28 (30.8)		21 (13.9)	196 (12.0)		0 (0.0)	0 (0.0)		0 (0.0)	1 (3.2)		2 (100.0)	5 (20.8)		4 (10.8)	22 (9.1)
60–69	2 (6.1)	8 (8.8)		8 (5.3)	98 (6.0)		0 (0.0)	0 (0.0)		0 (0.0)	0 (0.0)		0 (0.0)	0 (0.0)		4 (10.8)	12 (5.0)
≥ 70	1 (3.0)	6 (6.6)		5 (3.3)	53 (3.2)		0 (0.0)	0 (0.0)		0 (0.0)	0 (0.0)		0 (0.0)	0 (0.0)		4 (10.8)	8 (3.3)
**Total**	**33 (100.0)**	**91 (100.0)**		**151 (100.0)**	**1633 (100.0)**		**0 (0.0)**	**2 (100.0)**		**1 (100.0)**	**31 (100.0)**		**2 (100.0)**	**24 (100.0)**		**37 (100.0)**	**242 (100.0)**

Notes: Data show the number and proportion of patients with fatal and non-fatal carbon monoxide poisoning by sex and age group in the six districts of Ulaanbaatar where domestic use of raw coal was banned in May 2019 and replaced with refined coal briquettes, the three districts of Ulaanbaatar not subjected to the ban, and areas outside Ulaanbaatar.

In Ulaanbaatar’s districts without the ban, there were no fatal poisonings and one non-fatal poisoning before the ban, and two fatal and 31 non-fatal poisonings after the ban. Outside Ulaanbaatar, there were two fatal and 37 non-fatal poisonings before the ban, and 24 fatal and 242 non-fatal poisonings after the ban. The sex and age distribution of fatal and non-fatal poisonings were generally similar across the three settings. However, these data need to be interpreted with caution because the number of patients was small in the districts without the ban and outside Ulaanbaatar. 

**Fig. 1:**
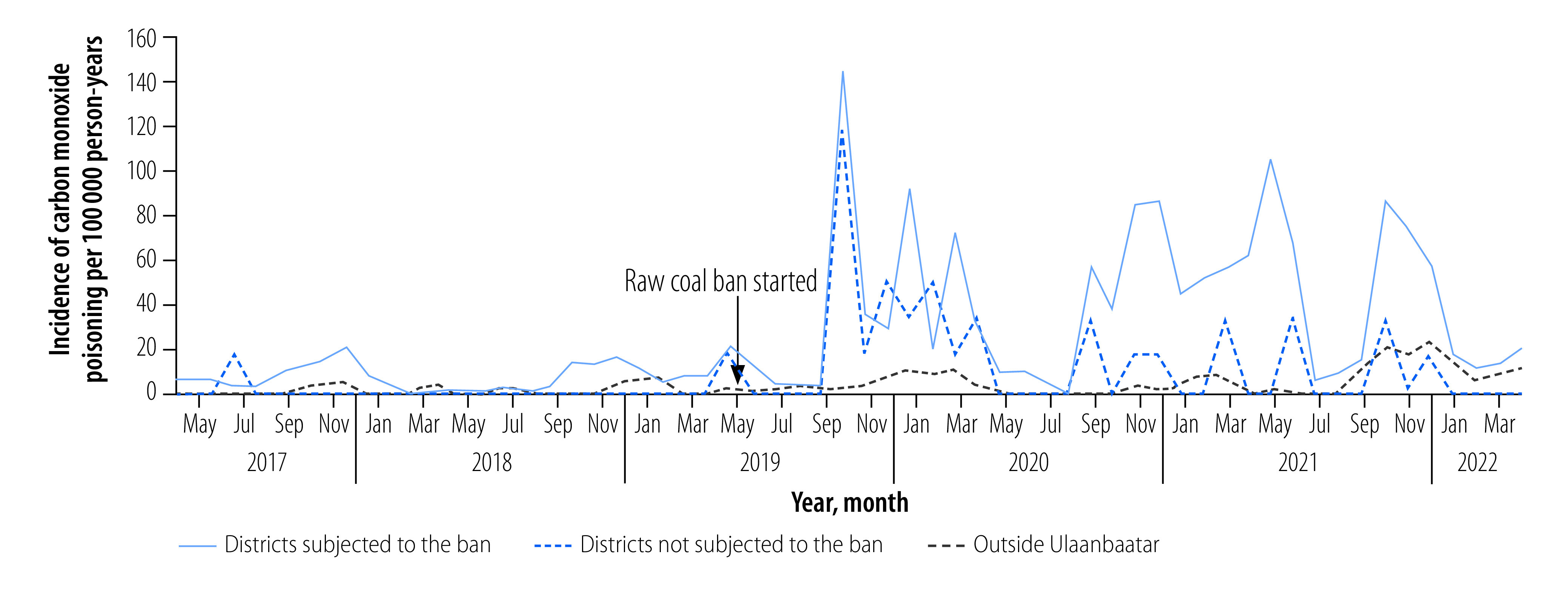
Monthly incidence of carbon monoxide poisoning before (May 2017 to April 2019) and after (May 2019 to April 2022) the raw coal ban in Ulaanbaatar, Mongolia

Notes: The data show the monthly incidence per 100 000 person-years of people with fatal and non-fatal carbon monoxide poisoning in the six districts of Ulaanbaatar where domestic use of raw coal was banned in May 2019 and replaced with refined coal briquettes, the three districts of Ulaanbaatar not subjected to the ban, and areas outside Ulaanbaatar.

Fig. 1 shows the trend in the monthly incidence of carbon monoxide poisoning in the three settings from May 2017 to April 2022. The ban was introduced in May 2019, and an abrupt increase in poisonings was recorded in Ulaanbaatar – not only in the districts with the ban but also in districts without the ban – in October 2019 when the cold season started. Afterwards, the incidence of poisoning fluctuated; however, it tended to be higher than that before the ban, especially in districts with the ban during the cold season. An abrupt increase in the incidence of poisoning after the ban was not observed outside Ulaanbaatar, but the incidence after the ban was slightly higher than before the ban during the cold season.

Table 2 shows the annual incidence of fatal and non-fatal carbon monoxide poisoning per 100 000 person-years by sex in 12-month periods before and after the ban. The overall incidence of carbon monoxide poisonings increased in districts with the ban, from 7.2 and 6.4 per 100 000 person-years in the two 12-month periods before the ban to 38.9, 42.0 and 40.1 per 100 000 in the three 12-month periods after the ban. The increase was observed among both sexes but more prominently for non-fatal carbon monoxide poisonings than for fatal cases. 

**Table 2:** Incidence of fatal and non-fatal carbon monoxide poisoning by sex in 12-month periods before and after the raw coal ban in Ulaanbaatar, Mongolia, 2017–2022

Setting and period	Male							Female					
	Population	Fatal poisonings			Non-fatal poisonings			Population	Fatal poisonings			Non-fatal poisonings	
		No. of cases	Incidence, per 100 000 person-years		No. of cases	Incidence, per 100 000 person-years			No. of cases	Incidence, per 100 000 person-years		No. of cases	Incidence, per 100 000 person-years
**Ulaanbaatar districts with the ban**													
Before ban													
May 2017–Apr 2018	641 738	17	2.6		33	5.1		696 519	6	0.9		40	5.7
May 2018–Apr 2019	660 318	8	1.2		29	4.4		709 186	2	0.3		49	6.9
After ban													
May 2019–Apr 2020	673 589	17	2.6		196	29.1		718 586	5	0.7		324	45.1
May 2020–Apr 2021	687 781	14	2.0		220	32.0		735 408	8	1.1		356	48.4
May 2021–Apr 2022	702 253	33	4.7		201	28.6		752 622	14	1.9		336	44.6
**Ulaanbaatar districts without the ban**													
Before ban													
May 2017–Apr 2018	34 104	0	0.0		1	2.9		35 879	0	0.0		0	0.0
May 2018–Apr 2019	34 691	0	0.0		0	0.0		36 136	0	0.0		0	0.0
After ban													
May 2019–Apr 2020	35 057	0	0.0		9	25.7		35 954	0	0.0		11	30.6
May 2020–Apr 2021	35 438	0	0.0		3	8.5		36 372	0	0.0		3	8.2
May 2021–Apr 2022	36 033	1	2.8		2	5.6		36 926	1	2.7		3	8.1
**Areas outside Ulaanbaatar**													
Before ban													
May 2017–Apr 2018	854 324	0	0.0		12	1.4		853 465	0	0.0		6	0.7
May 2018–Apr 2019	867 695	2	0.2		8	0.9		865 672	0	0.0		11	1.3
After ban													
May 2019–Apr 2020	869 763	3	0.3		26	3.0		867 217	3	0.3		38	4.4
May 2020–Apr 2021	875 796	3	0.3		15	1.7		873 081	0	0.0		14	1.6
May 2021–Apr 2022	886 142	11	1.2		62	7.0		882 628	4	0.5		87	9.9

Notes: Data are for the six districts of Ulaanbaatar where domestic use of raw coal was banned in May 2019 and replaced with refined coal briquettes, the three districts of Ulaanbaatar not subjected to the ban, and areas outside Ulaanbaatar.

Relative to the rate before the ban, the incidence rate ratios of fatal carbon monoxide poisoning among males were 1.3 (95% CI: 0.7–2.5), 1.1 (95% CI: 0.5–2.1) and 2.5 (95% CI: 1.4–4.3), respectively, in the first 12 months, 13–24 months and 25–36 months after the ban. The figures for females were 1.2 (95% CI: 0.3–4.2), 1.9 (95% CI: 0.6–5.8) and 3.3 (95% CI: 1.3–9.0), respectively, in the same time periods. The incidence rate ratios of non-fatal carbon monoxide poisoning among men were 6.1 (95% CI: 4.6–8.3), 6.7 (95% CI: 5.1–9.1) and 6.0 (95% CI: 4.5–8.1), respectively, in the same time periods. For females the incidence rate ratios of non-fatal poisonings in these time periods were 7.1 (95% CI: 5.6–9.1), 7.7 (95% CI: 6.1–9.8) and 7.1 (95% CI: 5.6–9.0), respectively.

In Ulaanbaatar districts without the ban, the number of fatal and non-fatal carbon monoxide poisonings before the ban was too small to calculate the incidence rate ratio. However, the number of non-fatal carbon monoxide poisonings increased in the first 12 months after the ban and decreased in the following months among both sexes. Outside Ulaanbaatar, the number of fatal carbon monoxide poisonings before the ban was too small to calculate the incidence rate ratio, but the number of poisonings increased after the ban among both sexes. The incidence rate ratios of non-fatal carbon monoxide poisonings outside Ulaanbaatar in the first 12 months, 13–24 months and 25–36 months after the ban were 2.6 (95% CI: 1.4–4.9), 1.5 (95% CI: 0.7–3.0) and 6.0 (95% CI: 3.6–10.5), respectively, among males, and 4.4 (95% CI: 2.4–8.4), 1.6 (95% CI: 0.7–3.5) and 10.0 (95% CI: 5.9–17.9), respectively, among females.

## Discussion

The incidence of domestic carbon monoxide poisoning increased abruptly in the districts of Ulaanbaatar where the domestic use of raw coal was banned and replaced with refined coal briquettes. The incidence of non-fatal carbon monoxide poisoning after the ban was over six times as high as before the ban among both sexes. The incidence of fatal carbon monoxide poisoning did not increase to the same extent. Despite public health measures to counteract carbon monoxide poisoning, the incidence of poisonings did not decrease. Notably, there was an abrupt increase in the incidence of non-fatal carbon monoxide poisoning in the first 12 months after the ban in the districts of Ulaanbaatar not subjected to the ban, although the incidence decreased afterwards. An increase in the incidence of non-fatal carbon monoxide poisoning was also observed outside Ulaanbaatar, although the increased incidence was much lower than that in the districts with the ban.

The large increase in the incidence of carbon monoxide poisoning in districts with the ban may be attributed to the nature and quality of briquettes, improper use of briquettes and furnaces and lack of ventilation. Compared with raw coal, briquettes require twice the amount of oxygen to burn, which might have led to incomplete combustion of briquettes in the closed furnaces, causing a build-up of carbon monoxide and soot.^21^ This process may be accelerated if briquettes are not well-dried.^22^ Then, soot agglomerates clog the air circulation in the furnace and chimneys, which might further accelerate incomplete combustion. Consequently, carbon monoxide leaks from the joints and cracks of furnaces and chimneys, and the carbon monoxide concentration increases inside the house owing to the lack of ventilation.^23^ In Mongolia, which has a long and very cold winter season, doors and windows are tightly closed, and ventilation is poor especially in traditional tent-like dwellings.

A question remains as to why the incidence of carbon monoxide poisoning abruptly increased after the ban in the districts without the ban. A probable reason is that residents were acquiring briquettes. For example, briquette factories were located in one of the districts without the ban. However, after the abrupt increase in carbon monoxide poisoning, the incidence decreased in the districts without the ban. This change is possibly because people in the districts without the ban could switch from briquettes to raw coal.

In the districts with the ban, non-fatal carbon monoxide poisoning increased shortly after the ban, but fatal carbon monoxide poisoning only increased during 25–36 months after the ban. In this period, there were 47 deaths due to carbon monoxide poisoning, of which 31 occurred from May to July 2021 when coronavirus disease 2019 (COVID-19) cases and deaths increased to a high level in Mongolia. The COVID-19 infection rates were < 1, 1108 and 26 840 cases per 100 000 people respectively in the three 12-month periods after the ban (May 2019 to April 2020; May 2020 to April 2021; May 2021 to April 2022). The mortality rates of COVID-19 were 0.0, 3.6 and 60.4 deaths per 100 000 respectively over the same time periods.^24^ The pandemic restrictions might also have prompted people to stay at home more than before, increasing the risk of carbon monoxide poisoning. This explanation might be supported by the increased carbon monoxide poisonings outside Ulaanbaatar during the same period.

The sex and age distributions of patients with carbon monoxide poisoning were almost identical before and after the ban, except for the age distribution of patients with fatal poisoning, in the districts with the ban. The proportion of adults among fatal cases after the ban was greater than that before the ban: 86% (78 adults) versus 70% (23 adults). The reason for this difference is unclear, but could be because the number of fatal poisonings before the ban was small.

Our study has some limitations. First, although the domestic use of raw coal was banned in the six study districts, some households might have continued using up their stocks of raw coal and therefore delayed the impact of the ban. However, the amount of raw coal stocked at home would probably have been limited and consumed shortly after the ban. Second, it is possible that carbon monoxide poisoning was underdiagnosed or was miscoded in the surveillance system. However, such errors, if any, would have occurred throughout the study period and we would not expect them to distort the results. Third, people suspected of carbon monoxide poisoning usually have their blood tested for confirmation, but some of them might have been misclassified as having COVID-19. Additionally, some people with suspected carbon monoxide poisoning might have avoided visiting health facilities, especially during the COVID-19 pandemic. Such misclassifications or health-seeking behaviours, if any, might have led us to underestimate the impact of the ban. However, we believe this possibility does not affect our conclusions, because we still saw an unusually high incidence of carbon monoxide poisoning during the pandemic.

In conclusion, we observed a large increase in the incidence of carbon monoxide poisoning after the raw coal ban in the districts of Ulaanbaatar subjected to the ban compared with other areas not subjected to the ban. Despite the government’s immediate response to the increased incidence of carbon monoxide poisoning, the incidence did not decrease, implying that improper use of coal briquettes and furnaces without sufficient ventilation is potentially widespread. To find effective methods of prevention of carbon monoxide poisoning, efforts are needed to investigate the current heating practice among households using briquettes, and to determine factors causing the high carbon monoxide concentration at home that can lead to carbon monoxide poisoning.
